# Modeling multi-stage disease progression and identifying genetic risk factors via a novel collaborative learning method

**DOI:** 10.1093/bioinformatics/btae728

**Published:** 2024-12-06

**Authors:** Duo Xi, Minjianan Zhang, Muheng Shang, Lei Du, Junwei Han, Alzheimer’s Disease Neuroimaging Initiative

**Affiliations:** School of Automation, Northwestern Polytechnical University, Xi'an 710072, China; School of Automation, Northwestern Polytechnical University, Xi'an 710072, China; School of Automation, Northwestern Polytechnical University, Xi'an 710072, China; School of Automation, Northwestern Polytechnical University, Xi'an 710072, China; School of Automation, Northwestern Polytechnical University, Xi'an 710072, China

## Abstract

**Motivation:**

Alzheimer’s disease (AD) typically progresses gradually for ages rather than suddenly. Thus, staging AD progression in different phases could aid in accurate diagnosis and treatment. In addition, identifying genetic variations that influence AD is critical to understanding the pathogenesis. However, staging the disease progression and identifying genetic variations is usually handled separately.

**Results:**

To address this limitation, we propose a novel sparse multi-stage multi-task mixed-effects collaborative longitudinal regression method (MSColoR). Our method jointly models long disease progression as a multi-stage procedure and identifies genetic risk factors underpinning this complex trajectory. Specifically, MSColoR models multi-stage disease progression using longitudinal neuroimaging-derived phenotypes and associates the fitted disease trajectories with genetic variations at each stage. Furthermore, we collaboratively leverage summary statistics from large genome-wide association studies to improve the powers. Finally, an efficient optimization algorithm is introduced to solve MSColoR. We evaluate our method using both synthetic and real longitudinal neuroimaging and genetic data. Both results demonstrate that MSColoR can reduce modeling errors while identifying more accurate and significant genetic variations compared to other longitudinal methods. Consequently, MSColoR holds great potential as a computational technique for longitudinal brain imaging genetics and AD studies.

**Availability and implementation:**

The code is publicly available at https://github.com/dulei323/MSColoR.

## 1 Introduction

Neurodegenerative disorders, such as Alzheimer’s disease (AD), progress gradually from prodromal symptoms to severe dementia (Knopman *et al.* 2021, Young *et al.* 2024). This dynamic process results in a high variance in disease biomarkers at different stages of the disease (Vogel *et al.* 2021). Therefore, longitudinal brain imaging scans encompassing multiple stages could provide valuable features for the accurate study of brain progression (Ching *et al.* 2023).

Brain imaging genetics has emerged as a crucial tool in brain science by analyzing associations between single nucleotide polymorphisms (SNPs) and quantitative traits (QTs) derived from neuroimaging scans (Shen and Thompson 2020). Since AD progression usually spans many years, assessing the changing pattern such as the baseline status and rates of change of longitudinal imaging QTs holds significant potential to characterize disease progression and provide valuable insights into AD. Ito *et al.* (2013) showed the significance of the baseline status in disease progression modeling, and Budgeon *et al.* (2017) demonstrated the effectiveness of the change rate of imaging QTs. Furthermore, Li *et al.* (2019) employed a mixed-effect model to consider slope and intercept, and Du *et al.* (2023) extended this method to incorporate genetic effects into AD progression modeling. However, these longitudinal methods often assume a constant rate of change, implicating a monotonically increasing or decreasing curve of changing pattern. In reality, patients with AD typically experience different rates of brain imaging changes due to the long-term effects of the disease (Young *et al.* 2024). This variability suggests that longitudinal imaging change rates may remain stable only for limited periods. For this reason, a single monotonic function is insufficient to capture the full progression of AD (Fortea *et al.* 2024). Thus, constructing a multi-stage model to analyze disease dynamics across different stages is meaningful and necessary (Chen *et al.* 2021).

With the above observations, jointly modeling multi-stage disease progression and identifying risk variants have the potential to enhance our understanding of AD. However, a major challenge is the limited availability of large, individual-level longitudinal imaging and genotype datasets due to privacy and long time-span constraints. To address these limitations, we integrate summary statistics from genome-wide association studies (GWAS) for brain imaging QTs of AD. GWAS typically involve large cohorts and provide single-SNP-single-QT associations for QTs of interest (Uffelmann *et al.* 2021), and yield valuable intermediate results for SNP-QT associations (Barbeira *et al.* 2018, Xi *et al.* 2023). Therefore, incorporating AD-oriented GWAS summary statistics into the joint model can enhance the multi-stage disease progression modeling and the identification of risk genetic variants.

**Figure 1.**
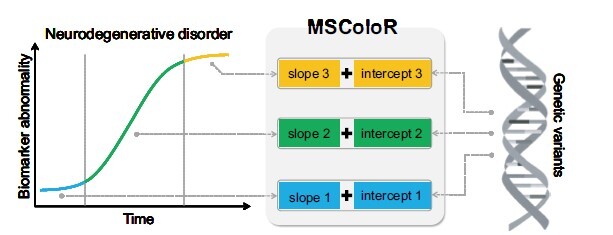
The typical conceptual illustration of the proposed method. This method models disease progress trajectory through slopes (indicating rates of change in longitudinal imaging, labeled as slopes 1, 2, and 3) and intercepts (representing baseline status, labeled as intercepts 1, 2, and 3) across multiple stages. The method aims to facilitate mutual enhancement between disease progression modeling and genetic risk factor identification.

In this article, we propose a novel multi-stage multi-task mixed-effects collaborative logitudinal regression method (MSColoR) to identify risk variations and model disease progression using longitudinal imaging data. Figure 1 presents the typical core concept of the MSColoR. Our method integrates GWAS summary statistics with a multi-stage mixed-effect model to improve multi-stage disease progression modeling and the identification of risk variants. To identify the most meaningful risk SNPs, we apply the $l_{\infty ,1}$ norm penalty (Gui *et al.* 2017, Zhao *et al.* 2021). Hence, our method has three distinct advantages: (i) We divide brain disorder progression into distinct phases, modeling each stage with its own slope and intercept, thereby avoiding the assumption of a constant progression rate. Importantly, the slope values for each stage can vary, and the number of stages is adjustable, enabling the model to accommodate various multi-stage diseases. (ii) We connect the genotype data to the intercept and slope of each disease stage, allowing us to simultaneously identify the genetic variations that contribute to multiple disease stages. (iii) We incorporate GWAS summary statistics through collaborative learning to address the challenges of small sample sizes of longitudinal imaging data. This method enhances the modeling of multi-stage disease trajectories and facilitates identifying genetic risk factors. Furthermore, we developed an efficient optimization algorithm with guaranteed convergence. Applying our method to both synthetic data with diverse characteristics and real data from the Alzheimer’s Disease Neuroimaging Initiative (ADNI) database, we demonstrate that MSColoR not only reduces modeling variance but also identifies meaningful genetic risk variations. In contrast, other state-of-the-art methods failed to identify some important loci, highlighting the superior performance of our method in identifying genetic risk factors. Additionally, the disease progression trajectories generated by our method outperform those of comparison models due to the more nuanced division of neurodegenerative disease progression. In conclusion, our approach provides a powerful and insightful framework for exploring the relationship between longitudinal brain imaging and genotype data, offering new potential for advancing brain imaging genetics.

## 2 Methods

### 2.1 GWAS for neuroimaging QTs

GWAS investigates the single-SNP-single-QT association via:

(1) $$y_{p}=\alpha +x_{g}\beta_{gp}+\varepsilon .$$

$x_{g}\in R^{n\times 1}$ and $y_{p}\in R^{n\times 1}$ denote the *g*th SNP and *p*th imaging QT of *n* subjects. $\beta_{gp}$ is the regression coefficient (effect size). $\alpha \in R^{n\times 1}$ is the intercept and $\varepsilon \in R^{n\times 1}$ represents the residual error. $\beta_{gp}=\frac{x_{g}^{T}y_{p}}{n-1}$ when SNP *g* and QT *p* have been normalized, and $\beta_{gp}$ is usually freely available.

This univariate GWAS model can be easily extended to the multivariate model where the effects of the whole genome are investigated. On this account, the effect size of all SNPs could be obtained by $u={\left(X^{T}X\right)}^{-1}X^{T}y={\hat{\Sigma }}_{XX}{}^{-1}\beta$, where $\beta \in R^{d\times 1}$ loads every SNP’s effect size of genotype **X** on **y**, which can be obtained from GWAS results. According to Yang *et al.* (2012), we can replace ${\hat{\Sigma }}_{XX}$ by $\Sigma_{XX}{}_{\text{ref}}/(n-1)$, where $X_{\text{ref}}$ can be obtained from a reference sample of the same ethnic group with individual-level genotype data. On this account, we can make use of the summary statistics from large GWAS to boost the performance of our proposed model in the following context.

### 2.2 One-stage longitudinal imaging genetic method

Sparse multitask mixed-effects longitudinal imaging genetic method (sMML) is developed to simultaneously fit the disease progression and identify risk genetic factors (Du *et al.* 2023). Assuming the effects of aging are consistent, $Z_{k}\in R^{n\times c}$ loads *c* imaging QTs of *n* subjects at the *k*th time point, $X\in R^{n\times d}$ is the genotype data with *d* SNPs, *t_k_* is the *k*th time point and *ϵ* is the error term, sMML takes the form:

(2) $$\begin{array}{c} \underset{W,V,A,B}{\min}\sum_{k=1}^{m}{\left\|Z_{k}-A-\mathrm{XW}-\left(B+\mathrm{XV}\right)t_{k}\right\|}_{F}^{2}+\Omega (W)+\Omega (V). \end{array}$$

$A\in R^{n\times c}$ and $B\in R^{n\times c}$ denote the population aging effects, including both baseline and rate of change for *c* imaging QTs. Here, XW and XV represent the intercept and slope effects induced by genetic factors for brain disorders of interest, where $W\in R^{d\times c}$ and $V\in R^{d\times c}$ loads regression coefficients accounting for the contribution of *d* SNPs to the intercept and slope corresponding to *c* imaging QTs.

However, the change rate of longitudinal brain imaging varies during the progression of neurodegenerative disorders like AD, and this single monotone decreasing or increasing function would be inadequate for disease progression modeling. Therefore, it is desired to develop a multi-stage model to investigate the changing patterns for different stages.

### 2.3 Multi-stage collaborative method

#### 2.3.1 Multi-stage longitudinal imaging genetic method

To more accurately model the disease progression trajectories and identify contributing genetic variations, we introduce a multi-stage multitask mixed-effects longitudinal method, defined as follows:

(3) $$\begin{array}{c} \underset{W,V,A,B}{\min}\sum_{s=1}^{L}\sum_{k=1}^{m}{\left\|Z_{ks}-A-XW_{s}-\left(B+XV_{s}\right)t_{ks}\right\|}_{F}^{2}+\Omega (W)+\Omega (V), \end{array}$$

where $Z_{ks}\in R^{n_{s}\times c}$ and $X_{s}\in R^{n_{s}\times d}$ denote the *c* imaging QTs and *d* genotype data of *n_s_* subjects at the *k*th time point of the *s*th disease stage. *L* is the number of total stages of the disease trajectory, $W=[W_{1},\ldots ,W_{L}]\in R^{d\times c\times L}$ and $V=[V_{1},\ldots ,V_{L}]\in R^{d\times c\times L}$ are parameter tensors denoted as intercepts and slopes of genetic effects of *L* stages, and $t_{ks}$ is the *k*th time point of the *s*th stage. **A** and **B** are the population aging effects, which enables our model to concentrate on the disease-related aspect.

#### 2.3.2 MSColoR with GWAS summary statistics

Since obtaining the individual-level longitudinal image and genetic data of large samples is challenging due to issues of privacy and time, we take two measures in this study. First, we use different sample sizes across different disease stages, as not all subjects are available at every time point. This strategy maximizes the sample size at each stage. Second, we leverage summary statistics from GWAS (samples >1000) to enhance the power of genetic factor identification. By collaborative learning, a better disease progression could be built. Consequently, we define the multi-stage multi-task mixed-effects collaborative longitudinal regression (MSColoR) as follows:

(4) $$\begin{array}{c} \underset{A,B,P,Q}{\min}\sum_{s=1}^{L}\sum_{k=1}^{m}{\left\|Z_{ks}-\gamma \left(X_{s}W_{s}+X_{s}V_{s}t_{ks}\right)-(1-\gamma )\left(A+Bt_{ks}\right)\right\|}_{F}^{2}\\ +\sum_{s=1}^{L}{\left\|y-X_{s}u_{s}\right\|}_{2}^{2}+\Omega (P)+\Omega (Q). \end{array}$$

In this equation, $P_{s}=\left[u_{s}\;W_{s}\right]\in R^{d\times (1+c)}$ and $Q_{s}=\left[u_{s}\;V_{s}\right]\in R^{d\times (1+c)}$ are joint parameter matrices for the *s*th stage, and $P=[P_{1},\ldots ,P_{L}]\in R^{d\times (1+c)\times L}$ and $Q=[Q_{1},\ldots ,Q_{L}]\in R^{d\times (1+c)\times L}$ are parameter tensors for all *L* stages. The second term incorporates GWAS summary statistics into the model, where $u_{s}$ is the corresponding effect size and can be obtained from GWAS results and a reference sample. Finally, to make our model more reasonable, we additionally use γ∈[0,1] to balance the contribution of the aging effect. To identify the most significant risk variants, we leverage $l_{\infty ,1}$ -norm to regularize **P** and **Q**, ie,

(5) $$\begin{array}{c} \Omega (P)=\lambda_{p}{\left\|P\right\|}_{\infty ,1}=\lambda_{p}\sum_{i=1}^{d}\underset{1\leq j\leq (c+1)}{\max}|P_{ij}|, \end{array}$$

where *λ_p_* is a nonnegative parameter to control the model’s sparsity. Ω(Q) is in the same form.

#### 2.3.3 Optimization

According to Eq. (4), our model is bi-convex in **A**, **B**, P, and Q. Thus, we solve these four parameters alternately.

- Solution of **A**: We first fix B,P and Q as constants, and then take the derivative with respect to **A** and set it to zero:
  (6) $$\begin{array}{c} -2(1-\gamma )\sum_{s=1}^{L}[\sum_{k=1}^{m}Z_{ks}-\gamma \left(mX_{s}W_{s}+\sum_{k=1}^{m}t_{ks}X_{s}V_{s}\right)]\\ +2{\left(1-\gamma \right)}^{2}\left(A+\sum_{s=1}^{L}\sum_{k=1}^{m}t_{ks}B\right)=0. \end{array}$$
  Solving this equation yields:
  (7) $$A=\frac{1}{\left(1-\gamma \right)}\sum_{s=1}^{L}\sum_{k=1}^{m}[Z_{ks}-\gamma \left(X_{s}W_{s}+X_{s}V_{s}t_{ks}\right)-(1-\gamma )Bt_{ks}].$$
  According to Eq. (7), we can attain the solution of **A** for all stages of disease development.
- Solution of **B**, P and Q: Similarly, we can solve **B** by
  (8) $$B=\sum_{s=1}^{L}\sum_{k=1}^{m}[[Z_{ks}-\gamma \left(X_{s}W_{s}+X_{s}V_{s}t_{ks}\right)-(1-\gamma )A]/t_{ks}].$$

Since P is composed of $u_{s}$ and $W_{s}$, we separately solve them. We take the derivative of Eq. (4) for each $W_{s}$, set it to zero, and handle them based on the Karush–Kuhn–Tucker (KKT) conditions. This yields the solution to $W_{s}$ as:

(9) $$W_{s}=\frac{\gamma \sum_{k=1}^{m}X_{s}^{T}[Z_{ks}-\gamma X_{s}V_{s}t_{ks}-(1-\gamma )\left(A+Bt_{ks}\right)]}{\gamma^{2}X_{s}^{T}X_{s}+\lambda_{ps}D_{ps}}.$$

In this equation, $D_{ps}$ is a diagonal matrix whose *i*th element is $\frac{1}{2\max\left|p^{i}\right|}(i=1,\ldots ,d)$.

Based on the same procedure, we can solve each $V_{s}$ as

(10) $$V_{s}=\frac{\gamma \sum_{k=1}^{m}t_{ks}X_{s}^{T}[Z_{ks}-\gamma X_{s}W_{s}-(1-\gamma )\left(A+Bt_{ks}\right)]}{\gamma^{2}\sum_{k=1}^{m}t_{ks}^{2}X_{s}^{T}X_{s}+\lambda_{qs}D_{qs}},$$

where $D_{qs}$ is a diagonal matrix with the *i*th diagonal entry being $\frac{1}{2\max\left|q^{i}\right|}(i=1,\ldots ,d)$.

Finally, $u_{s}$ depends on $P_{s}$ and $V_{s}$, and can be calculated by:

(11) $$\begin{array}{c} u_{s}=\frac{X_{s}^{T}y}{X_{s}^{T}X_{s}+\lambda_{ps}D_{ps}+\lambda_{qs}D_{qs}}=\frac{\beta }{{\hat{\Sigma }}_{XX}+\lambda_{ps}{\tilde{D}}_{ps}+\lambda_{qs}{\tilde{D}}_{qs}}, \end{array}$$

where ${\tilde{D}}_{ps}$ and ${\tilde{D}}_{qs}$ are diagonal matrices with their *i*th elements are $\frac{1}{2(n-1)\max\left|p^{i}\right|}(i=1,\ldots ,d)$ and $\frac{1}{2(n-1)\max\left|q^{i}\right|}(i=1,\ldots ,d)$.

Depending on the above closed-form solutions, the final solution can be generated through iterative optimization. We present the pseudocode of the optimization in Algorithm 1. According to Eq. (7), (8), (9) and (10), we alternately handle four convex sub-problems. Therefore, the objective value will monotonously decrease during the iteration.

Algorithm 1. The MSColoR algorithm

**Require:** The genotype data $X\in R^{n\times d}$, longitudinal imaging QTs $Y\in R^{n\times c\times L\times m}$ for *L* stages, GWAS summary statistics $\beta \in R^{d\times 1}$, and ${\hat{\Sigma }}_{XX}$ estimated from the reference sample. The parameters $\lambda_{ps}$ and $\lambda_{qs}$.

**Ensure:**  A,B, P, and Q.

1: Initialize $B\in R^{n\times c},W\in R^{d\times c}\;\text{and}\;V\in R^{d\times c}$;

2: **while** not convergence **do**

3:   Solve **A** by Eq. (7), and **B** by Eq. (8);

4:   Update ${\tilde{D}}_{ps}$ and ${\tilde{D}}_{qs}$, and solve each $u_{s}$ by Eq. (11);

5:   Update $D_{ps}$, and solve each $W_{s}$ by Eq. (9);

6:   Update $D_{qs}$, and solve each $V_{s}$ by Eq. (10);

7: **end while**

## 3 Experimental results

### 3.1 Validation method

As no existing multi-stage multitask longitudinal imaging genetics methods are available for direct comparison, we developed three closely related methods for evaluation. Of note, our method has two objectives: modeling multi-stage disease progression and identifying genetic variations, whereas some of the comparison methods address only one of these goals.

- Sparse multitask regression method (sMTR) (Wang *et al.* 2012): sMTR jointly models the correlation between SNPs and longitudinal imaging QTs at each time point: $\underset{W}{\min}\sum_{k=1}^{m}{\left\|Y_{k}-XW_{k}\right\|}_{F}^{2}+\lambda {\left\|W\right\|}_{2,1}$, where $Y_{k}\in R^{n\times c}$ is the imaging matrix at *k*th time point, $W=[W_{1},\ldots ,W_{m}]\in R^{d\times c\times m}$. This method focuses on identifying risk genetic variations and cannot build the disease progression.
- Sparse multitask mixed-effects longitudinal imaging genetic method (sMML, Section 2.2): sMML is a reduced version of MSColoR, which considers a one-stage disease progression, i.e. $\underset{W,V,A,B}{\min}\sum_{k=1}^{m}{\left\|Z_{k}-A-\mathrm{XW}-\left(B+\mathrm{XV}\right)t_{k}\right\|}_{F}^{2}+\lambda_{1}{\left\|W\right\|}_{2,1}+\lambda_{2}{\left\|V\right\|}_{2,1}$.
- Sparse multi-stage multitask mixed-effects longitudinal imaging genetic method (MSMML): this is a another control version of our method without using summary statistics. This can help investigate the contribution of summary statistics, i.e. $\underset{A,B,P,Q}{\min}\sum_{s=1}^{L}\sum_{k=1}^{m}{\left\|Z_{ks}-\gamma \left(X_{s}W_{s}+X_{s}V_{s}t_{ks}\right)-(1-\gamma )\left(A_{s}+B_{s}t_{ks}\right)\right\|}_{F}^{2}+\lambda_{1}{\left\|P\right\|}_{\infty ,1}+\lambda_{2}{\left\|Q\right\|}_{\infty ,1}$.

### 3.2 Evaluation criteria and hyperparameters

The root mean square error (RMSE) is used to evaluate the degree of model fitting. The RMSE for the *j*th imaging QT for subject *i* is defined as: ${\text{RMSE}}_{ij}=\sqrt{\frac{1}{n}{\left\|y_{ij}-y_{ij}\right\|}_{2}^{2}}$. We showed the average RMSE across all imaging QTs and subjects. A smaller RMSE value indicates a better performance.

To find the most appropriate hyperparameters, we employed the nested 5-fold cross-validation. The internal loop aimed to find appropriate parameters, and the outside loop generated the final results. Specifically, we set a candidate set in a moderate interval $10^{i}\left(i=[-3,-2,\ldots ,2,3]\right)$ since too large and too small parameters will lead to undesirable biomarkers. Meanwhile, to ensure efficiency, we employed $\max_{j}\left|w_{j}^{\left(t+1\right)}-w_{j}^{\left(t\right)}\right|\leq \epsilon$ and $\max_{j}\left|v_{j}^{\left(t+1\right)}-V_{j}^{\left(t\right)}\right|\leq \epsilon$ as additional stopping criteria, where *ϵ* was the tolerable error and empirically set to $10^{-5}$ in this paper.

All methods were carried out on the same software platform and used the same data partition to ensure the fairness of comparison.

### 3.3 Results on synthetic data

All findings on synthetic data (Supplementary Note A) indicate that our method effectively models longitudinal imaging genetics by dividing disease progression into multiple stages and leveraging GWAS summary statistics.

### 3.4 Results on real neuroimaging genetic data

#### 3.4.1 Data source

##### 3.4.1.1 Individual-level neuroimaging genetic data of ADNI

Brain imaging and genetic data were obtained from the ADNI database (adni.loni.usc.edu). A key objective of ADNI is to evaluate whether serial MRI, alongside other biomarkers, and clinical and neuropsychological assessment, can be combined to track the progression of mild cognitive impairment and early AD. For the latest information, see www.adni-info.org.

Neuroimaging and genetic data for non-Hispanic Caucasian subjects were downloaded. Longitudinal imaging QTs were derived from MRI T1-weighted scans at five time points: baseline (BL), month 6 (M06), month 12 (M12), month 24 (M24), and month 36 (M36). The scans from each subject were aligned across these time points, and individuals missing genotype or imaging data at any point were excluded. The demographic information for each stage is summarized in Table 1. To study disease trajectories of different stages, the 3-year data were divided into three stages: stage 1 (BL, M06, and M12), stage 2 (M06, M12, and M24), and stage 3 (M12, M24, and M36), as shown in Supplementary Table S2. We used the FreeSurfer software to extract the cortical thickness values of regions of interest. Details of this preprocessing protocol can be found in the previously reported work (Shen *et al.* 2010). In practice, only disease-associated imaging QTs are useful to identify genetic factors. Therefore, we selected 12 FreeSurfer imaging QTs as biomarkers for AD, as shown in Supplementary Table S3. Additionally, some other covariates such as gender, education, and handedness were removed from the imaging QTs because they may influence brain status and potentially misguide the identification of genetic effects.

**Table 1.** Participant characteristics at baseline.

	HC	MCI	AD
Number	180	259	133
Gender (M/F)	99/81	173/86	74/59
Handedness (R/L)	167/13	236/23	125/8
Age (mean ± SD)	76.05 ± 4.98	75.11 ± 6.96	75.30 ± 7.62
Education (mean ± SD)	16.20 ± 2.65	15.88 ± 3.00	14.90 ± 3.09

The genotypic data from the ADNI database first underwent quality control and imputation. We analyzed all 145 124 SNPs of chromosome 19, as it contains the well-known AD risk genes such as *APOE*, *APOC1*, and *TOMM40*. This study has two primary objectives: identifying relevant SNPs contributing to AD progression and establishing a plausible multi-stage progression trajectory of AD.

##### 3.4.1.2 AD-related GWAS summary statistics

We employed a GWAS study with respect to AD which contained 94,437 non-Hispanic white individuals, including 35, 274 ADs cases and 59 163 controls (Kunkle *et al.* 2019). In this study, we used 145, 124 SNPs’ GWAS effect sizes of chromosome 19.

In addition, we employed the EUR data of the 1000 Genome Consortium Project (1kGP) (release 20130521) to estimate the genetic covariance Σ_*XX*_. The 1kGP database collected genetic variations of diverse ethnic populations (1kG Project Consortium 2015), allowing researchers to use other ethnic groups based on their specific research needs.

#### 3.4.2 Improved degree of model fitting

The average RMSEs of MSColoR and three comparison methods on this dataset are presented in Table 2, including both training and testing RMSEs. Since sMTR and sMML model only one-stage disease trajectory, we calculated the significance *P*-value between their RMSEs and the corresponding average RMSEs of our three-stage method. MSColoR obtained smaller RMSEs than the other three methods, indicating superior modeling performance over sMTR, sMML, and MSMML. Although RMSEs of MSMML in the third stage were not significantly different from those of MSColoR, MSMML missed AD-related factors in this stage (Section 3.4.3). Overall, these findings suggest that leveraging summary statistics from large GWAS, utilizing the multi-stage strategy of the disease progression, and removing the impact of normal aging, may better capture the pathology and etiology of AD.

**Table 2.** RMSEs (mean  ±  SD) on ADNI data set.^a^

	Training	Testing
sMTR	10.0230 ± 0.1036****	9.8512 ± 0.2439****
sMML	0.8577 ± 0.0532****	1.0578 ± 0.0661***
MSMML		
Stage 1	0.6608 ± 0.0030****	0.7788 ± 0.0535^ns^
Stage 2	0.6586 ± 0.0047****	0.8626 ± 0.0980*
Stage 3	0.6510 ± 0.0087**	1.0483 ± 0.0660^ns^
MSColoR		
Stage 1	0.6212 ± 0.0014	0.7989 ± 0.1106
Stage 2	0.6262 ± 0.0029	0.7579 ± 0.0539
Stage 3	0.6351 ± 0.0078	1.0245 ± 0.1672

a Statistical differences (*t*-test) between comparison methods and MSColoR are presented. ns: not significant, *: *P* ≤ 0.05, **: *P* ≤ 0.01, ***: *P* ≤ 0.001 , ****: *P* ≤ 0.0001.

#### 3.4.3 Identification of genetic factors

**Figure 2.**
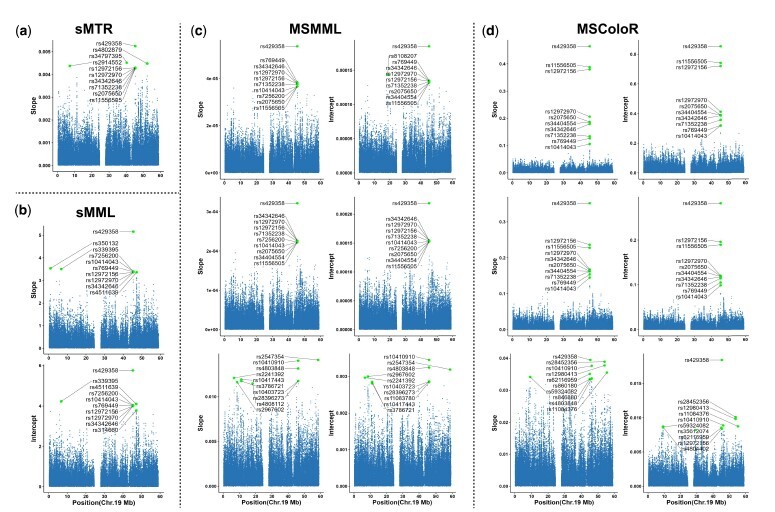
Mean canonical weights for SNPs of 5-fold cross-validation trials. Except for sMTR, the other comparison methods and our methods present two rows/columns for slope and intercept results respectively. Since (a) sMTR and (b) sMML are one-stage models, they only have one-stage subfigure, respectively. For (c) MSMML and (d) MSColoR, there are three rows for three disease stages, respectively.

Identifying meaningful risk variants is also crucial for understanding AD. In Fig. 2, we presented scatter plots of SNP weight coefficients for MSColoR and three comparison methods. The *y*-axis in each sub-plot represents the average effect of each SNP, while the *x*-axis shows the genomic position. Higher *y*-axis values indicate a stronger SNP effect. For clarity, the top 10 SNPs with the highest weights in each subfigure are marked and annotated. Notably, all four methods identified the well-established risk locus rs429358 (*APOE*). In Supplementary Fig. S2 (Appendix), we provided locus plots for rs429358, identified at each stage for both slope and intercept of MSColoR. These plots reveal that rs429358 is a missense variant in the *APOE* gene on chromosome 19, consistently showing the highest significance in association with AD. However, MSMML missed this star risk locus in stage 3, and only one of its top 10 SNPs was AD-related. In contrast, MSColoR outperformed MSMML by leveraging summary statistics, which enhanced its performance as though running on a larger sample size. Importantly, the top 10 SNPs of the first two stages of MSColoR are all AD risk SNPs, highlighting MSColoR’s superior ability to identify risk variants. Further analysis of the rank order of the top ten SNPs across all three stages for MSMML and MSColoR provided additional insights. Three AD risk loci, including rs12972156, rs11556505, and rs34342646, within the *NECTIN2* gene, maintained the same order for both methods during the first two stages. Additionally, rs12972156 consistently ranked higher in the second stage than in the first, while the opposite trend was observed for rs2075650 (*TOMM40*). These results suggest that MSColoR accurately identifies AD risk variants and captures their varying significance across different disease stages, indicating that the impact of risk loci evolves as the disease progresses. The scatter plots further show that baseline status and rate of change are underpinned by distinct sets of genetic factors, emphasizing the importance of considering both measures for AD. In summary, by segmenting disease progression into multiple stages and incorporating GWAS summary statistics, MSColoR effectively identifies AD risk genetic factors and reveals their dynamic roles in disease progression.

### 3.5 Effectiveness of genetic risk factors

To validate the statistical significance of the identified genetic variants, we applied a one-way analysis of variance (ANOVA) to assess the influence of the top ten SNPs on diagnostic status at each stage, with age, gender, handedness, and years of education included as covariates. A locus was considered effective if its main effect on the diagnostic status was significant. The *P*-values for the top 10 SNPs identified by slopes and intercepts at all three stages, obtained from the one-way ANOVA, are summarized in Supplementary Table S4, showing that almost all *P*-values were statistically significant (<0.05). Specifically, the *P*-values for all top 10 variants identified by both slope and intercept were significant in the first two stages. Even in the third stage, most *P*-values for SNPs in these top 10 SNPs are statistically significant, particularly for the intercept. This demonstrates that MSColoR can effectively identify AD risk variants by jointly modeling multi-stage disease development and finding out risk genetic variations. Furthermore, by incorporating staged disease progression trajectory information, MSColoR can more accurately and comprehensively identify disease risk variants.

### 3.6 Improved fitting of disease progression

**Figure 3.**
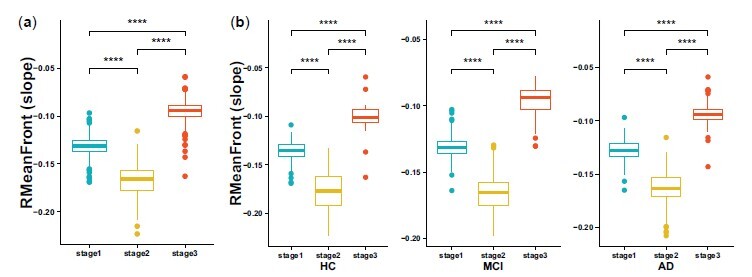
Fitted disease progression for different diagnostic groups in terms of slope (rate of progression) of MSColoR. (a) Results for all subjects, while (b) for subjects in different diagnostic groups. Statistical results (*t*-test) between different diagnostic groups are presented. The three sub-figures in each panel are slopes or intercepts corresponding to QT RMeanFront. ns: not significant, *P ≤ 0.05, **P ≤ 0.01, ***P ≤ 0.001, ****P ≤ 0.0001.

To present fitted disease progression, we displayed the distributions of the fitted slopes at different stages (stages 1, 2, and 3) for QT RMeanFront in Fig. 3a. Results for both slopes and intercepts for all 12 QTs are shown in Supplementary Fig. S3a (Appendix). As anticipated, significant differences between stages suggest effective capture of complex disease progression. As expected, all statistical results reached the significance level (*P* < 0.05). For more details, we present the fitted slopes of QT RMeanFront across different disease stages for each diagnostic group in Fig. 3b, along with the statistical differences between stages. Results for both slopes and intercepts for all 12 QTs are shown in Supplementary Fig. S3b (Appendix). The consistently negative slopes indicate a steady decline in AD progression, aligning with prior findings that cortical thickness decreases continuously throughout AD progression (Brouwer *et al.* 2022). Significant differences were observed between stages, with the rate of decline in the second stage notably faster than in the first and three. This pattern, evident in most imaging QTs, suggests a marked acceleration in AD progression, underscoring the value of a multi-stage model for fitting disease trajectories. Compared to MSMML (see Supplementary Fig. S4 in the Appendix), MSColoR provided a superior fit for disease progression, emphasizing the importance of incorporating GWAS summary statistics. Overall, our multi-stage collaborative learning method offers a promising alternative for disease progression modeling.

### 3.7 Effectiveness of disease progression trajectory

Given that cognitive scores are clinically valid assessments for high-risk populations, we investigated the correlations between slope and cognitive scores, as well as those between the intercept and cognitive scores. Specifically, we applied five cognitive scores to indicate cognitive levels, i.e. the Mini Mental State Examination (MMSE) score, the Alzheimer’s disease assessment scale–cognitive subscale (ADAS-cog) score and three Rey’s Auditory Verbal Learning Test (RAVLT) scores. Additionally, we also examined the slope’s and intercept’s correlations to the diagnostic status. For demonstration purposes, we averaged the slopes and intercepts across multiple brain regions and investigated their correlations with cognitive scores and diagnostic status. The correlation coefficients and their significance level (*P*-values) are presented in Table 3. All correlation coefficients for both slope and intercept at the three stages were statistically significant. These results demonstrate that MSColoR effectively models the disease progression trajectory, indicating its potential utility for downstream tasks and clinical practice.

**Table 3.** Correlation coefficients between disease progression and five types of cognitive scores and the diagnostic status at each disease stage.^a^

	Stage 1	Stage 2	Stage 3
MMSE			
Slope	0.3365****	0.5085****	0.4232****
Intercept	0.3122****	0.4913****	0.4165****
ADAS			
Slope	0.3286****	0.4995****	0.2921****
Intercept	0.3035****	0.4852****	0.2887****
RAVLT_Tol^b^			
Slope	0.3555****	0.4084****	0.3034****
Intercept	0.3337****	0.3940****	0.3042****
RAVLT_30^c^			
Slope	0.3173****	0.3311****	0.2573***
Intercept	0.3044****	0.3168****	0.2575***
RAVLT_Rec^d^			
Slope	0.2358****	0.3123****	0.3074****
Intercept	0.2203****	0.3067****	0.3077****
Diagnosis			
Slope	0.3714****	0.4369****	0.2748****
Intercept	0.3553****	0.4249****	0.2749****

a The asterisks denote the significance level. *: *P* ≤ 0.05, **: *P* ≤ 0.01, ***: *P* ≤ 0.001, ****: *P* ≤ 0.0001.

b Total score of the first five learning trials.

c 30-minute delay total number of words recalled.

d 30-minute delay recognition score.

## 4 Discussion

The brain structure of individuals with neurodegenerative diseases, especially AD, typically follows three stages (Therriault *et al.* 2024). The first stage exhibits subtle changes with little or no cognition decline, the second stage changes rapidly due to the combined effect of AD attack and normal aging, and the third stage changes gradually due to the combined effect at the final period of AD and aging (Fortea *et al.* 2024, Therriault *et al.* 2024). To model this time-varying disease progression, we proposed a multi-stage learning method that does not require a uniform sample size across stages. This maximizes the sample size at each stage and thus alleviates the sample size limitation that longitudinal data usually faces. Our results showed smaller RMSEs compared to one-stage methods (considering only one progression stage for AD with a long cycle) in both simulated and real datasets (Sections 3.4.2 and Supplementary Note A.2). The fitted slopes and intercepts of imaging QTs across stages (Section 3.6) showed significant differences. Moreover, our method also identifies risk genetic factors varied across the three stages (Section 3.4.3), implicating a distinct genetic basis for different progression stages. Additionally, we found significant correlations between the disease progression and five AD-related cognitive scores, including MMSE, ADAS, RAVLT_Tol, RAVLT_30, and RAVLT_Rec, as well as diagnostic status (Section 3.7). Notably, the slope values in our model are independent for each stage and can vary, allowing flexibility in pre-modeling to determine the optimal number of stages. By adjusting the value of L of Eq. (3), our model can accommodate disease progressions with more or fewer than three stages, making it adaptable to various multi-stage disease progressions.

While MSColoR exhibits significant advantages in joint modeling AD progression and identifying genetic risk factors, several limitations warrant further discussion and consideration. First, the model performance is somewhat enhanced by using large GWAS summary statistics. And thus suitable disease-specific summary statistics may not be available and must be sourced from publicly accessible results. Second, MSColoR integrates longitudinal neuroimaging-derived QTs and SNPs. However, the longitudinal imaging QTs may be not available for the disease of interest. Future directions may incorporate multiple cohorts to imitate longitudinal data and validate its applicability across different populations and imaging modalities. In summary, MSColoR demonstrates promising capabilities by addressing the limitations of existing longitudinal methods and shows its utility in brain imaging genetics.

## 5 Conclusion

Neurodegenerative disorders such as AD typically progress gradually, yet most existing methods model longitudinal imaging data using a constant monotonic function, neglecting the potential for dynamic disease trajectories. Furthermore, identifying the genetic basis is crucial for understanding disease pathogenesis. In this paper, we propose a novel sparse MSColoR. MSColoR segments long-term disease progression into multiple stages and collaboratively identifies genetic risk variations using large GWAS results. We evaluated our method on both synthetic and real ADNI datasets, where MSColoR outperformed three benchmark methods, achieving lower RMSEs and identifying more accurate and meaningful genetic variations. Additionally, the distributions of fitted slopes and intercepts across diagnostic groups and the correlations between disease progression trajectories and cognitive scores or diagnostic status at different stages demonstrate that MSColoR effectively models AD progression. Future work could extend the applicability of our method to other chronic diseases or developmental disorders.

## Supplementary data

Supplementary data are available at *Bioinformatics* online.

Conflict of interest: None declared.

## Funding

This work was supported in part by the MOST 2030 Brain Project Grant (No. 2022ZD0208500), part by the National Natural Science Foundation of China (Nos. 61936007, 62136004, 62373306, U23A20335, and 61973255), and part by the Fundamental Research Funds for the Central Universities at Northwestern Polytechnical University.

## Supplementary Material

btae728_Supplementary_Data

## Data Availability

The data underlying this article are available in the article and in its online supplementary material.
