# Supplementary material for: Modeling multi-stage disease progression and identifying genetic risk factors via a novel collaborative learning method
Source: Bioinformatics. 2024 Dec 6;41(1):btae728. doi: 10.1093/bioinformatics/btae728 (PMC11784593; doi:10.1093/bioinformatics/btae728)
Supplement: btae728_Supplementary_Data [file btae728_supplementary_data.pdf]

Supplementary Materials for "Modeling multi-stage disease progression and identifying genetic risk factors via a novel collaborative learning method" by Duo Xi, Minjianan Zhang, Muheng Shang, Lei Du, and Junwei Han.

A. Supplementary Note: Results on synthetic data

A.1. Data source

In the simulation, we generated two longitudinal imaging datasets with 3 stages of disease progression and each stage had 3 time-points. We first used the real genotype data with 6,000 SNPs of 267 subjects and then normalized it to have zero mean and 0.05 standard deviation. The k-th imaging data at time-point j of the s-th stage was constructed by:

$$\mathbf{Z}_{ks} = \gamma\left(\mathbf{X}_s\mathbf{W}_s + \mathbf{X}_s\mathbf{V}_s t_{ks}\right) + (1-\gamma)\left(\mathbf{A} + \mathbf{B}t_{ks}\right) + \epsilon, \tag{S1}$$

where $\mathbf{W}_s$ and $\mathbf{V}_s$ represented the ground truth of the baseline status and change rate of the disease progression at stage k. The error term $\epsilon \sim N(o, \sigma)$ followed a normal distribution with mean zero and standard deviation σ. $\mathbf{A}$ and $\mathbf{B}$ denoted the aging effect for both controls and cases. In this study, $c = 4$, $\sigma = 1$, and $t = [0, 0.5, 1; 0.5, 1, 2; 1, 2, 3]$ for three stages.

A.2. Improved degree of model fitting

We presented RMSEs in Table S1. Our MSColoR obtained smaller RMSEs than all comparison methods on both training and testing sets. Specifically, for all four methods, sMTR presented the biggest RMSEs on training and testing sets, suggesting the advantage of building the disease progression and disentangling the influence of aging. Moreover, compared with sMTR or sMML, both multi-stage methods, i.e., MSMML and MSColoR had smaller RMSEs, indicating the advancement of dividing the disease into multiple stages. Besides, our method obtained better RMSEs than MSMML. These results demonstrated that using GWAS summary statistics showed a higher fitting degree of the longitudinal data.

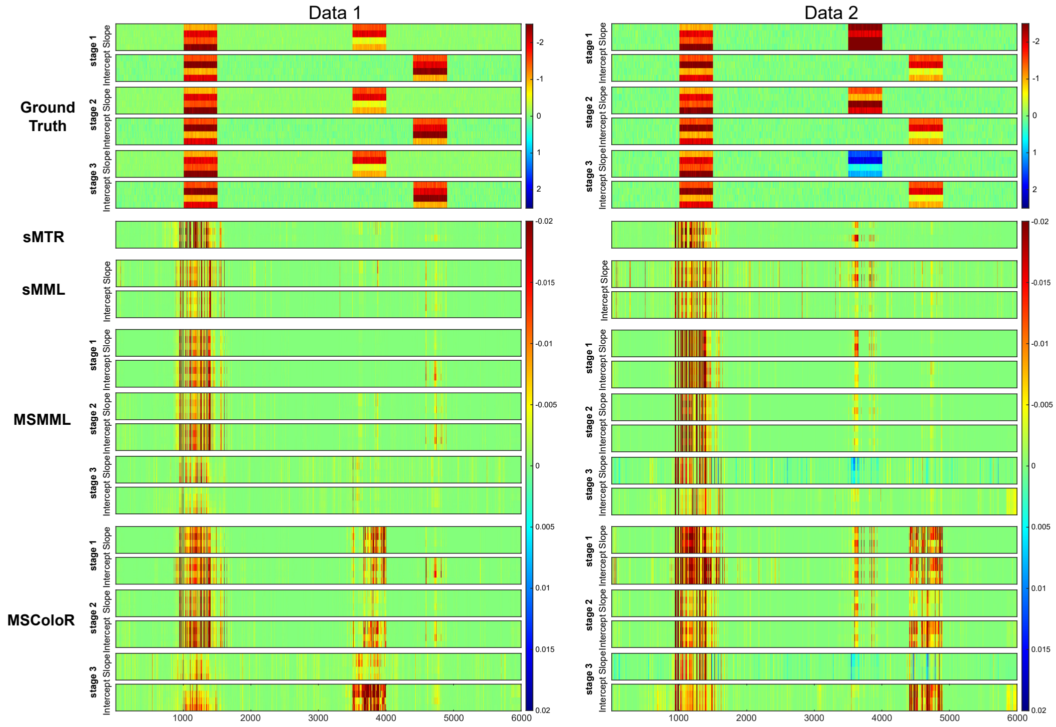

Fig. S1: Canonical weights on simulation data. Rows from top to bottom: Ground truth (slope) and Ground truth (intercept) for three stages, sMTR, sMML (slope), sMML (intercept), MSMML (slope), and MSMML (intercept) for all three stages, MSColoR (slope) and MSColoR (intercept) for all three stages respectively.

A.3. Identification of related underlying factors

Fig. S1 showed the weight values of the slope and intercept for each stage. We also presented true underlying factors for the slope and intercept of the three stages for clarity. Since sMTR could not model the disease progression, there was only one weight vector. sMTR and sMML were one-stage methods, and there was only one result for them. In this figure, MSColoR performed better in identifying the risk factors than comparison methods because they missed some underlying factors. For example, the second data simulated different

progression rates for three stages. sMTR and sMML were unable to recover such information, but MSMML and MSColoR can identify this pattern correctly. Moreover, when compared to MSMML, MSColoR can also identify weaker signals ignored by MSMML with the help of GWAS summary statistics.

In summary, the above findings suggested that our method can fit quite well for longitudinal imaging genetics by removing the impact of normal aging, dividing disease progression into multiple stages, and leveraging GWAS summary statistics.

B. Supplementary Tables

Table S1. RMSEs (mean ± std) on simulation data sets. Statistical comparison (t-test) between comparison methods and MSColoR are presented in parentheses. ns: not significant, *: $p \leq 0.05$, **: $p \leq 0.01$, ***: $p \leq 0.001$, ****: $p \leq 0.0001$.

		Data 1		Data 2	
		Training	Testing	Training	Testing
sMTR		2.1454±0.0276 (****)	2.1586±0.0327 (****)	1.9074±0.0331 (****)	1.9166±0.0435 (****)
sMML		0.5614±0.0261 (****)	0.5655±0.0496 (****)	0.6011±0.0212 (****)	0.6065±0.0438 (****)
MSMML	stage 1	0.4109±0.0061 (***)	0.4207±0.0100 (**)	0.5738±0.0065 (****)	0.5825±0.0082 (***)
	stage 2	0.4219±0.0315 (**)	0.4314±0.0631 (ns)	0.4987±0.0089 (****)	0.5284±0.0206 (****)
	stage 3	0.3263±0.0274 (ns)	0.3579±0.0202 (ns)	0.4575±0.0508 (**)	0.4640±0.0682 (ns)
MSColoR	stage 1	0.3826±0.0091	0.3975±0.0137	0.4554±0.0113	0.4945±0.0391
	stage 2	0.3688±0.0139	0.3892±0.0095	0.3927±0.0164	0.4300±0.0127
	stage 3	0.3165±0.0240	0.3550±0.0242	0.3750±0.0210	0.4106±0.0329

Table S2. Participant characteristics at the first time point of each disease stage.

	stage 1			stage 2			stage 3		
	HC	MCI	AD	HC	MCI	AD	HC	MCI	AD
Number	177	228	133	157	120	107	122	71	23
Gender (M/F)	99/78	150/78	74/59	82/75	81/39	63/44	65/57	55/16	16/7
Handedness (R/L)	164/13	210/18	125/8	145/12	113/7	99/8	112/10	65/6	20/3
Age (mean±std)	76.00±5.01	75.39±7.06	75.30±7.62	75.90±4.96	75.09±6.58	75.45±7.13	75.44±4.94	75.14±6.83	74.54±5.42
Education (mean±std)	16.21±2.65	15.93±2.99	14.90±3.09	116.24±2.66	16.11±288	14.91±3.11	16.16±2.68	16.00±2.87	15.17±2.57

Table S3. Volumetric/thickness measures-derived imaging QTs used in this paper.

QT ID	ROI
LHippVol<br>RHippVol	Volume of hippocampus
Lparahipp<br>Rparahipp	Thickness of parahippocampal gyrus
LEntCtx<br>REntCtx	Thickness of entorhinal cortex
LPrecuneus<br>RPrecuneus	Thickness of precuneus
LMeanFront<br>RMeanFront	Mean thickness of caudal midfrontal, rostral midfrontal, superior frontal, lateral orbitofrontal, medial orbitofrontal gyri and frontal pole
LMeanLatTemp<br>RMeanLatTemp	Mean thickness of inferior temporal, middle temporal, and superior temporal gyri

Table S4. Top ten SNPs selected by MSColoR at each disease stage.

**stage 1**				**stage 2**				**stage 3**			
slope		intercept		slope		intercept		slope		intercept	
SNPs	*p*-value	SNPs	*p*-value	SNPs	*p*-value	SNPs	*p*-value	SNPs	*p*-value	SNPs	*p*-value
rs429358	1.84E-14	rs429358	1.84E-14	rs429358	2.81E-13	rs429358	2.81E-13	rs429358	1.72E-04	rs429358	1.72E-04
rs11556505	2.54E-09	rs11556505	2.54E-09	rs12972156	7.17E-09	rs12972156	7.17E-09	rs28452356	7.45E-01	rs28452356	7.45E-01
rs12972156	1.30E-09	rs12972156	1.30E-09	rs11556505	1.48E-08	rs11556505	1.48E-08	rs12980413	7.27E-01	rs12980413	7.27E-01
rs12972970	1.30E-09	rs12972970	1.30E-09	rs12972970	7.17E-09	rs12972970	7.17E-09	rs10410910	1.41E-01	rs10410910	1.41E-01
rs2075650	2.54E-09	rs2075650	2.54E-09	rs34342646	7.17E-09	rs2075650	1.48E-08	rs11084376	4.36E-01	rs11084376	4.36E-01
rs34404554	2.54E-09	rs34404554	2.54E-09	rs2075650	1.48E-08	rs34404554	1.48E-08	rs62116959	3.80E-01	rs59324082	1.48E-01
rs34342646	1.30E-09	rs34342646	1.30E-09	rs34404554	1.48E-08	rs34342646	7.17E-09	rs59324082	1.48E-01	rs4804402	2.82E-01
rs71352238	1.30E-09	rs71352238	1.30E-09	rs71352238	7.17E-09	rs71352238	7.17E-09	rs4803848	1.33E-01	rs62116959	3.80E-01
rs769449	2.53E-12	rs769449	2.53E-12	rs769449	2.76E-11	rs769449	2.76E-11	rs860180	5.13E-01	rs12972156	4.24E-02
rs10414043	7.51E-13	rs10414043	7.51E-13	rs10414043	1.21E-11	rs10414043	1.21E-11	rs846880	5.13E-01	rs35612074	1.74E-02

C. Supplementary Figures

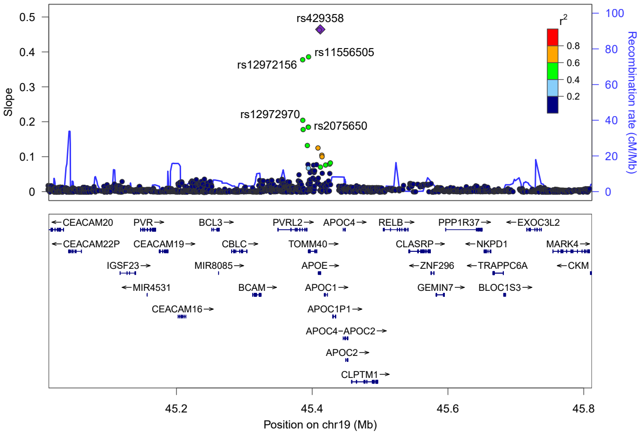

(a) Locus plot for slope at the first stage

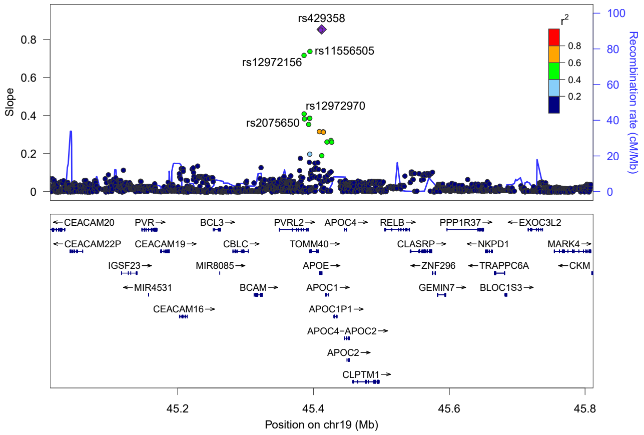

(b) Locus plot for intercept at the first stage

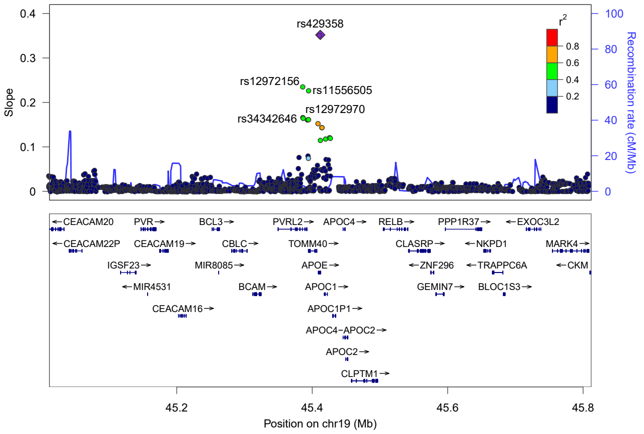

(c) Locus plot for slope at the second stage

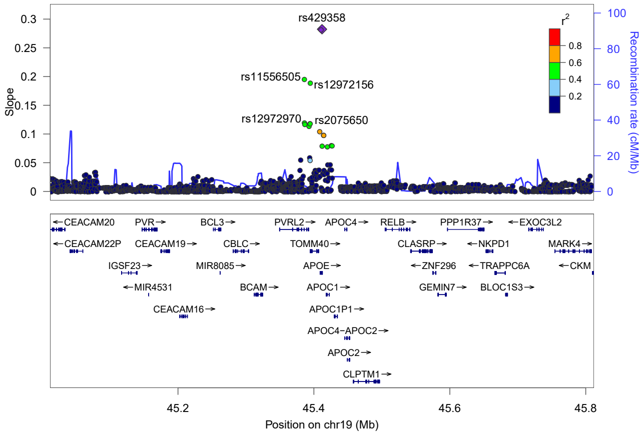

(d) Locus plot for intercept at the second stage

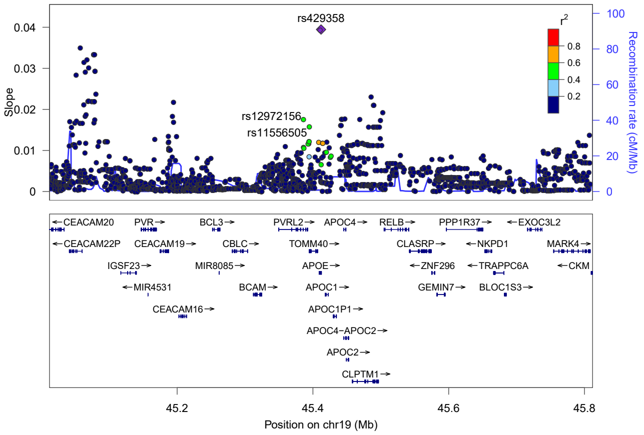

(e) Locus plot for slope at the third stage

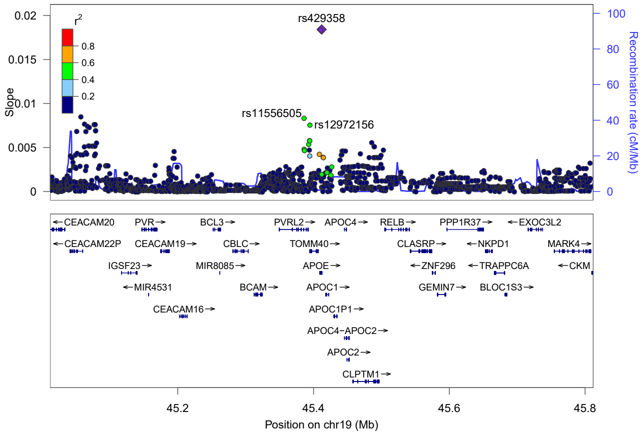

(f) Locus plot for intercept at the third stage

Fig. S2: Locus plots for the top lead SNPs for slope (rate of progression) and intercept (baseline status) at each stage.

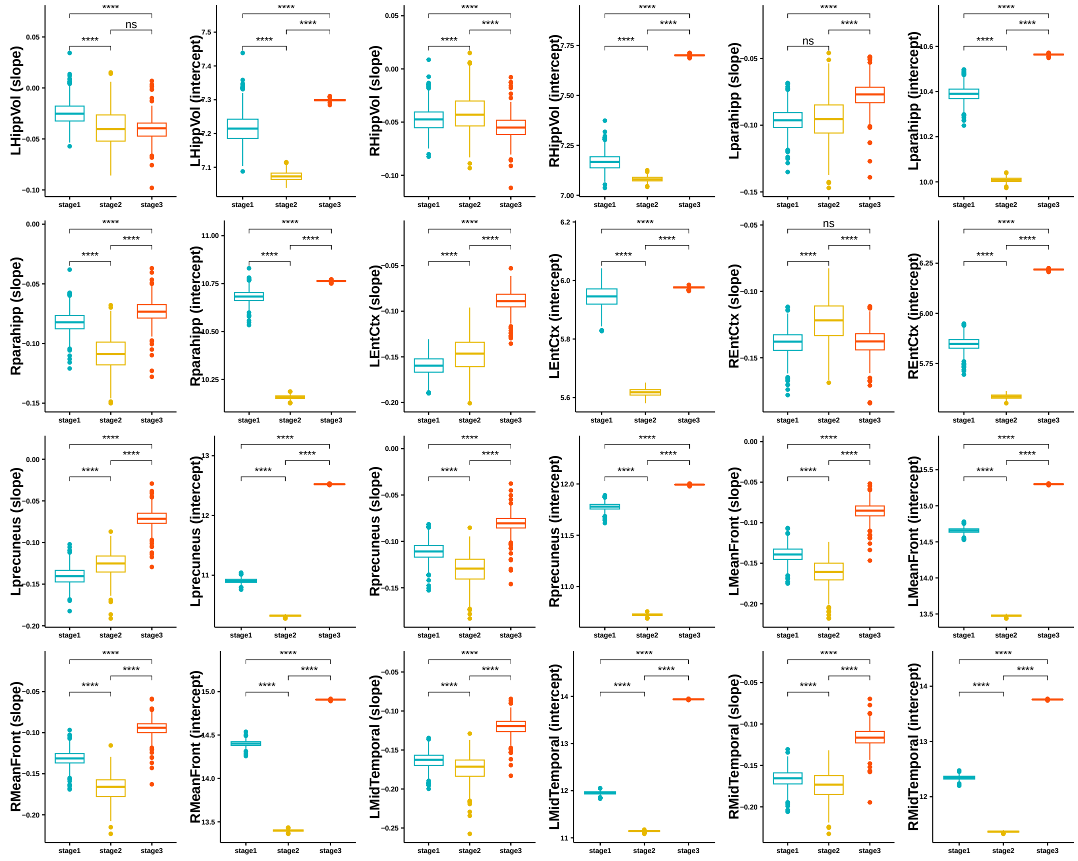

(a) Fitted disease progression for different diagnostic groups

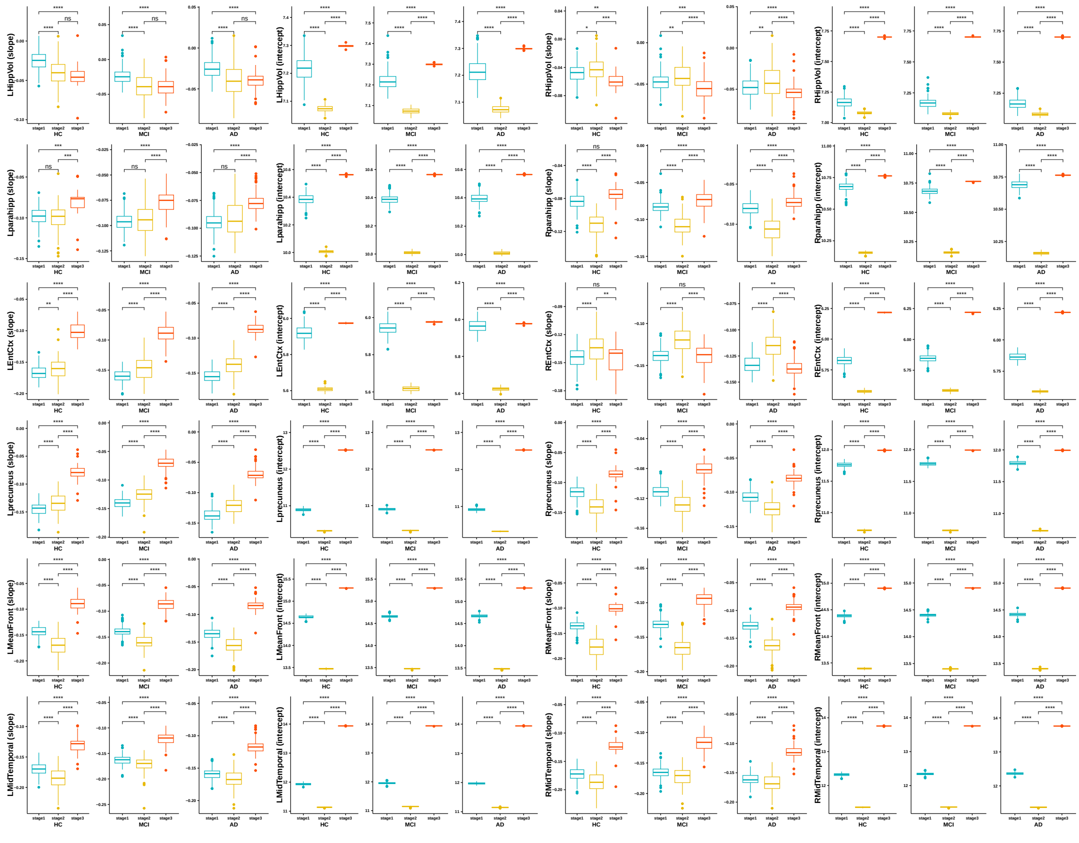

(b) Fitted disease progression for different disease stages within each diagnostic group

Fig. S3: Intercept (baseline status) and slope (rate of progression) of MSColoR. Statistical results (t-test) between different stages are presented. In each row, every one (a) or three (b) sub-figures are slopes or intercepts corresponding to one QT. ns: not significant, *: $p \leq 0.05$, **: $p \leq 0.01$, ***: $p \leq 0.001$, ****: $p \leq 0.0001$.

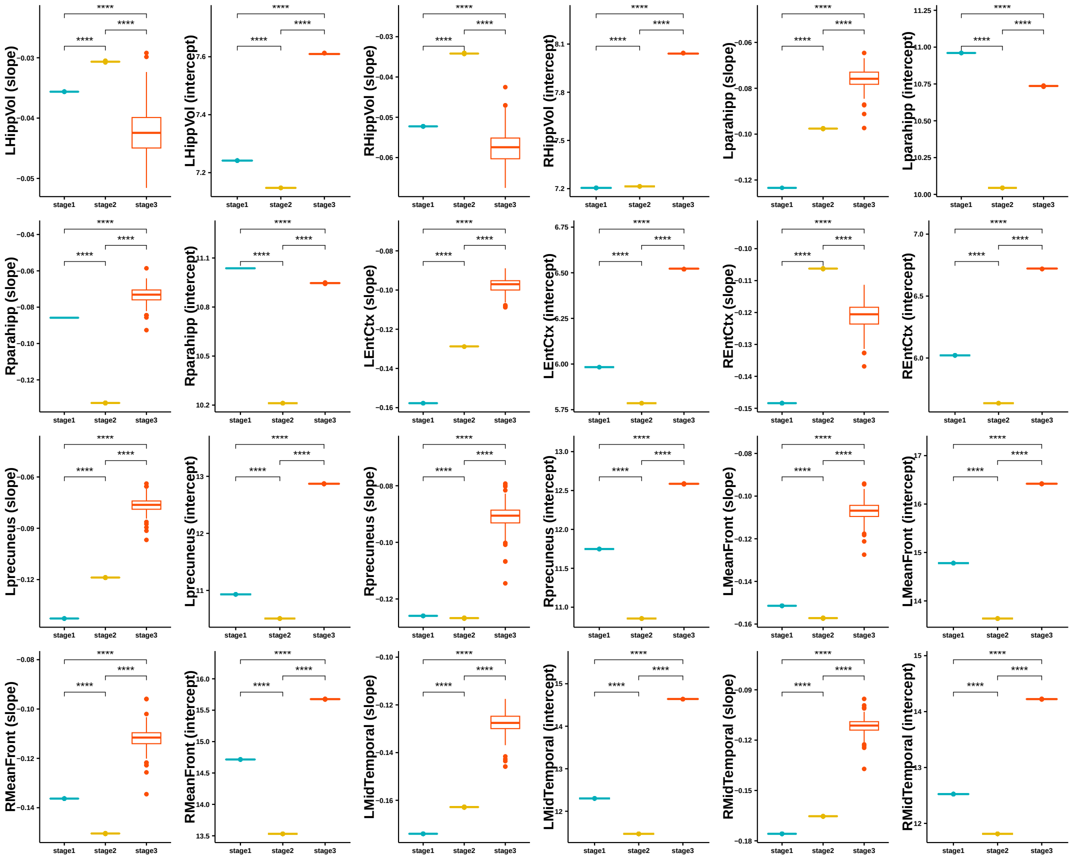

(a) Fitted disease progression for different diagnostic groups

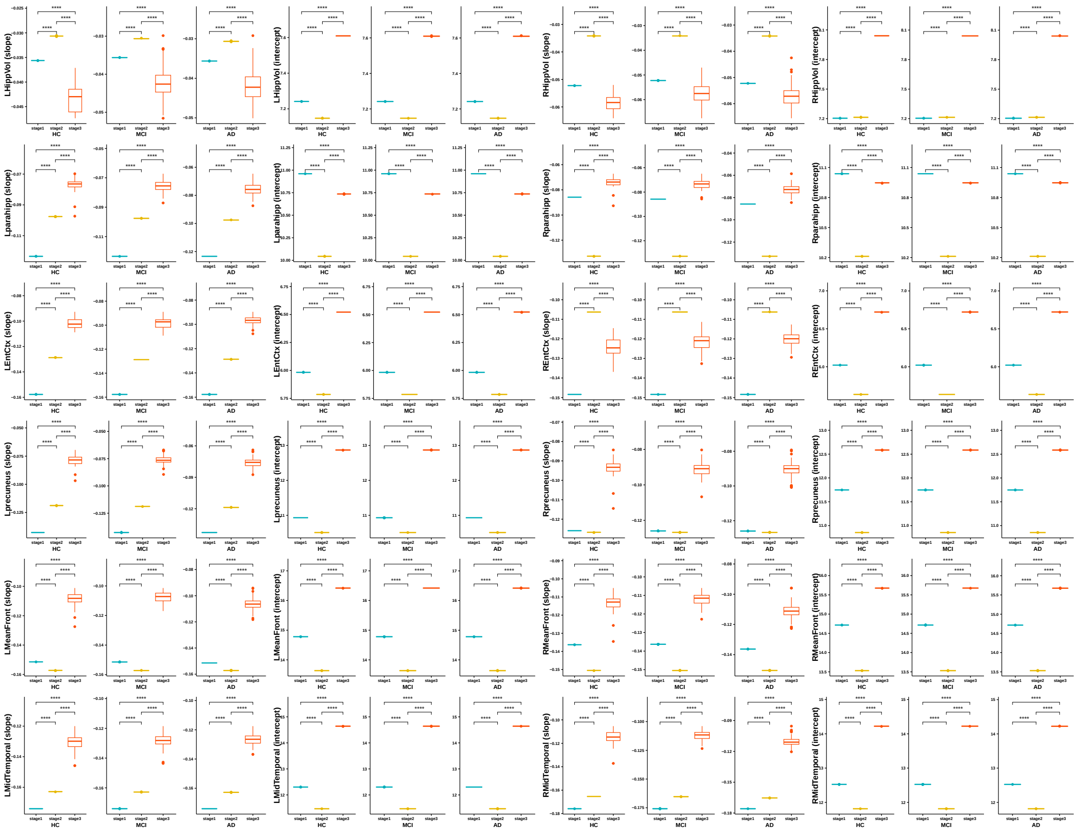

(b) Fitted disease progression for different disease stages within each diagnostic group

Fig. S4: Visualization of intercept (baseline status) and slope (rate of progression) estimated by MSMML. Statistical differences (t-test) between stages are presented. In each row, every one (a) or three (b) sub-figures are the results of slope or intercept related to one QT. ns: not significant, *: $p \leq 0.05$, **: $p \leq 0.01$, ***: $p \leq 0.001$, ****: $p \leq 0.0001$.
